# Immunoglobulin G N-glycosylation remodeling in viral infections: immunomodulatory roles and potential biomarker implications

**DOI:** 10.3389/fimmu.2026.1758338

**Published:** 2026-02-05

**Authors:** Ruxu Yan, Chunqing Wang, Xuezhen Zhao, Yingjie Wang, Meng Liu, Xiaohong Shi, Hanxiang Chen, Dong Li

**Affiliations:** 1 Department of Clinical Laboratory Medicine, Shandong Medicine and Health Key Laboratory of Laboratory Medicine, The First Affiliated Hospital of Shandong First Medical University & Shandong Provincial Qianfoshan Hospital, Jinan, China; 2 School of Public Health, Shandong First Medical University & Shandong Academy of Medical Sciences, Jinan, China; 3 School of Public Health, Jining Medical University, Jining, China

**Keywords:** virus infection, IgG N-glycosylation, galactosylation, sialylation, fucosylation

## Abstract

The glycosylation of Immunoglobulin G (IgG), a complex post-translational modification, is essential for the structure and function of IgG. The varying affinities of different IgG N-glycans for Fcγ receptors can influence inflammatory responses, including antibody-dependent cell-mediated cytotoxicity (ADCC), complement-dependent cytotoxicity (CDC), and antibody-dependent cell phagocytosis (ADCP). Existing research indicates a correlation between IgG N-glycosylation and a range of diseases, such as autoimmune disorders, neurologic diseases, and cancer. Persistent and emerging viral infections pose a significant risk to public health, and the elicitation of innate and adaptive immune responses in the host following viral infection is intricately linked to inflammatory mediators, yet the relationship between viral infections and IgG N-glycosylation has not been systematically explored. In this review, we delineate the typical immune response to viral infection, expound on the structure and functionality of IgG N-glycans, and summarize the alterations in IgG N-glycans in human serum/plasma post-viral infection, along with the underlying inducements for these modifications. These alterations could serve as biomarkers for viral infectious diseases, thereby facilitating early detection and prognostic assessment of such conditions. Furthermore, exploring these modifications may elucidate the molecular mechanisms underlying the diseases and identify novel therapeutic targets.

## 1 Introduction

Viral infections are a significant concern for public health, as viruses, regardless of their genetic and capsid structures, play pivotal roles in human infections and diseases. Hepatitis B virus (HBV) and hepatitis C virus (HCV) infect approximately 295.9 million and 57.8 million people worldwide, respectively, and are major inducers of liver cancer (1). Around 38 million people worldwide are living with human immunodeficiency virus (HIV). Persistent HIV infection induces immune activation, chronic inflammation, multiorgan damage (2). Since the emergence of the Coronavirus Disease 2019 (COVID-19), it has rapidly evolved from a localized issue into a global pandemic. This epidemic presents unprecedented challenges to the global public health infrastructure and exerts profound impacts on the socio-economic landscape and individual lifestyles (3). The threat to global public health is exacerbated by numerous viral infectious diseases for which vaccines, antiviral treatments, and diagnostic markers are often lacking, particularly for newly emerging viruses (4). Consequently, understanding immune responses to viral infections and developing antiviral strategies can help reduce the spread and impact of these pathogens.

Viral infections elicit inflammation and immune responses-processes pivotal to antiviral defense. Upon viral invasion, the innate immune system (encompassing macrophages, NK cells, and the secretion of proinflammatory cytokines) first orchestrates an immediate, non-antigen-specific defense cascade to constrain early viral replication and dissemination (5). This innate response further drives the activation of the adaptive immune system: professional antigen-presenting cells (e.g., dendritic cells) mature upon sensing viral components, process and present viral epitopes to T lymphocytes. Activated T cells (especially follicular helper T cells, Tfh) in turn activate B lymphocytes via Cluster of Differentiation 40 and its ligand Cluster of Differentiation 40 Ligand (CD40-CD40L) interaction and cytokine secretion (6). As a core effector molecule of adaptive immunity, immunoglobulin G (IgG) fulfills indispensable roles in antiviral defense. IgG participates in antiviral immune responses through multiple mechanisms, including virus neutralization, phagocytosis promotion, and complement system activation (7). Additionally, the interaction between the crystallizable fragment (Fc) of IgG and Fcγ receptors is critical for antibody-mediated effector functions, which, together with antigen-binding fragments (Fab) segment-mediated viral neutralization, mobilizes effector cells to participate in immune responses and enhances antiviral defense (8).

IgG function is tightly regulated by N-glycosylation, a post-translational modification essential for its immune activities; distinct glycosylation patterns can drive divergent immune response outcomes (9). Over recent decades, IgG N-glycosylation has emerged as a critical correlate of multiple pathologies: studies demonstrate its high sensitivity in distinguishing healthy individuals from patients with autoimmune diseases, infectious diseases, and cancer (10). The N-glycosylation profile of IgG exhibits variability across different diseases, indicating its potential utility as a specific biomarker (11). Notably, alterations in IgG N-glycosylation have also been observed after infections with bacterial and parasitic pathogens. For instance, IgG1 galactosylation was associated with Mycobacterium tuberculosis infection, while changes in fucosylation patterns may affect IgG-mediated clearance of Plasmodium (12, 13). In addition to IgG, immunoglobulins such as Immunoglobulin A (IgA), Immunoglobulin M (IgM), and Immunoglobulin E (IgE) can also undergo N-glycosylation modifications, which are associated with certain pathogenic infections. For example, sialylation of IgA has been reported to correlate with disease severity in both COVID-19 and influenza A (14, 15). Additionally, increased levels of high-mannose and sialylated IgM isoforms are linked to greater severity of COVID-19 in a separate investigation (16). While these studies have progressively elucidated the importance of alterations in immunoglobulin glycosylation modifications in the onset and progression of various diseases, comprehensive research on glycosylation modifications of immunoglobulins other than IgG in various pathological conditions remains limited. Furthermore, considering that IgG is the most abundant immunoglobulin in human serum and serves as a crucial effector molecule in humoral immunity, this review focuses on the investigation of IgG N-glycosylation.

This article investigates the intrinsic connection between viral infections and immune responses, with a particular emphasis on recent research on alterations in IgG N-glycosylation in human serum or plasma during infections, and systematically elucidates the critical role of various IgG N-glycosylation modifications in disease progression and immune regulation.

## 2 Viral infection and immunological reaction

Millions of deaths occur annually due to viral infections, including chronic viral pathogens (e.g., HBV, HCV) and emerging zoonotic viruses (e.g., COVID-19, influenza A/H5N1) (17, 18). These viral infections, whether chronic or acute, impose a major global public health burden. Upon viral invasion, the host initiates a sequentially activated and synergistically coordinated immune response, in which the innate immune system acts as the first line of defense. This rapid-acting response is mediated by pattern recognition receptors (PRRs) expressed on innate immune cells (e.g., macrophages, dendritic cells, and natural killer cells) (19). PRRs conservatively recognize conserved pathogen-associated molecular patterns (PAMPs) derived from viral components (e.g., viral RNA, glycoproteins) and damage-associated molecular patterns (DAMPs) released by infected or stressed cells (20). Engagement of PRRs triggers downstream inflammatory signaling cascades (e.g., NF-κB, IRF pathways), leading to the production of pro-inflammatory cytokines (interleukin (IL)-6, tumor necrosis factor (TNF)-α), chemokines (CCL2, CXCL10), and type I interferons (IFN-I) (21, 22). These soluble mediators not only directly inhibit viral replication and spread but also recruit and activate other immune cells, thereby laying the foundation for the subsequent adaptive immune response.

The adaptive immune response is activated following antigen presentation initiated by the innate immune response. Professional antigen-presenting cells (APCs), primarily dendritic cells (DCs), phagocytose viral particles or infected cells, process viral antigens into peptide fragments, and present these peptides on major histocompatibility complex (MHC) class I and class II molecules (23). MHC class I-presented viral peptides are recognized by CD8+ T cells, which require two signals for activation and differentiation into cytotoxic T lymphocytes (CTLs): the first signal is the binding of T cell receptor (TCR) to the MHC-I-peptide complex, and the second signal is provided by DC-derived co-stimulatory molecules and cytokines (24). Once activated, CTLs specifically recognize and eliminate virus-infected cells by releasing perforin and granzyme, or by inducing apoptosis via the Fas-Fas ligand (FasL) pathway. Concurrently, MHC class II-presented peptides are recognized by CD4+ T cells, which differentiate into specialized T helper (Th) cell subsets (e.g., Th1, Th2, Tfh cells) depending on the cytokine microenvironment. Tfh cells migrate to germinal centers (GCs) in secondary lymphoid organs, where they interact with B cells through cell-cell contact (e.g., CD40-CD40L) and secretion of cytokines (e.g., IL-21, IL-4), providing essential signals for B cell activation and maturation (25, 26).

**Figure 1:**
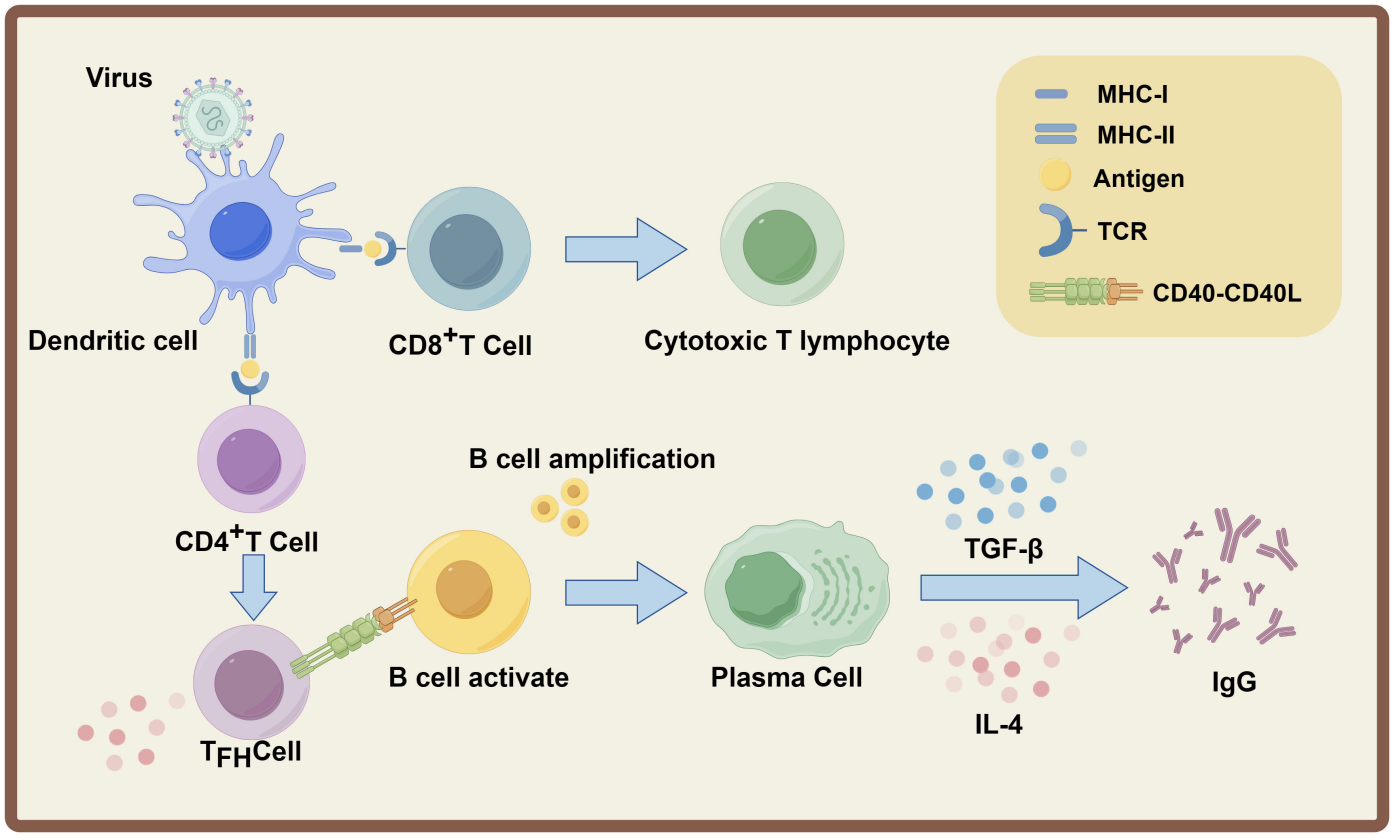
Schematic illustration of the adaptive immune response induced by viral infection. Cellular immunity: Dendritic cells (DCs) present endogenous viral antigen peptides via major histocompatibility complex (MHC) class I to CD8^+^ T cells; activated CD8^+^ T cells differentiate into CTLs to clear infected cells. Humoral immunity: DCs present exogenous viral antigen peptides via MHC-II to CD4^+^ T cells, which differentiate into Tfh cells upon co-stimulation and cytokines. Tfh cells activate B cells via TCR-MHC-II recognition, CD40-CD40L interaction and IL-4; activated B cells proliferate into plasma cells that first secrete IgM, then mainly IgG after antibody class switching under cytokine regulation.

Activated B cells undergo clonal expansion and differentiate into either short-lived plasmablasts or long-lived plasma cells, which secrete large quantities of antigen-specific antibodies. Additionally, a subset of B cells differentiates into memory B cells, enabling rapid and robust immune responses upon re-exposure to the same virus (27). Among the five antibody isotypes (IgM, IgG, IgA, IgD, IgE), IgG is the predominant isotype in the late phase of the systemic primary immune response and the entire systemic secondary immune response, while secretory IgA (sIgA) dominates the mucosal secondary immune response (28). IgG production is tightly regulated by Tfh cell-derived cytokines and B cell-intrinsic signaling pathways (29). The resulting IgG antibodies exert multiple antiviral effects, including neutralization of viral particles (preventing viral entry into host cells), activation of the complement system (mediating lysis of infected cells), and antibody-dependent cellular cytotoxicity (ADCC) or phagocytosis (recruiting NK cells or macrophages to eliminate infected cells) (30, 31). Collectively, B cell-mediated humoral immunity (predominantly IgG secretion) and T cell-mediated cellular immunity (predominantly CTL activity) are core effector mechanisms of the adaptive immune response, which synergistically contribute to the clearance of viral infections and the establishment of long-term protective immunity (**Figure 1**).

## 3 Structure and function of IgG N-glycans

IgG, an abundant immunoglobulin in human serum, is a Y-shaped glycoprotein composed of four polypeptide chains: two identical heavy chains (H chains) and two identical light chains (L chains). Papain cleavage of IgG at the hinge region yields two identical Fab and Fc (32). The Fab region, containing the variable light (VL), variable heavy (VH), constant light (CL) and constant heavy 1 (CH_1_) domains, mediates antigen binding, while the Fc region, consisting of the constant heavy 2 (CH_2_) and constant heavy 3 (CH_3_) domains of the two heavy chains, interacts with cell surface Fc receptors (FcRs) and does not bind antigens (33). Glycosylation, a sophisticated post-translational modification of proteins, regulates protein stability, folding, cellular trafficking, signaling transduction, and interactions with other macromolecules (34). Protein glycosylation is categorized into N-linked (occurring on asparagine residues) and O-linked (occurring on serine or threonine residues) glycosylation (35). IgG O-linked glycosylation is challenging to characterize due to its heterogeneous core structures and the absence of conserved consensus sequences (36). In contrast, N-linked glycosylation of IgG has been extensively investigated and well characterized. A conserved N-linked glycosylation site at asparagine 297 (Asn297) of the IgG Fc region critically modulates the structure and function of the Fc domain (37).

**Figure 2:**
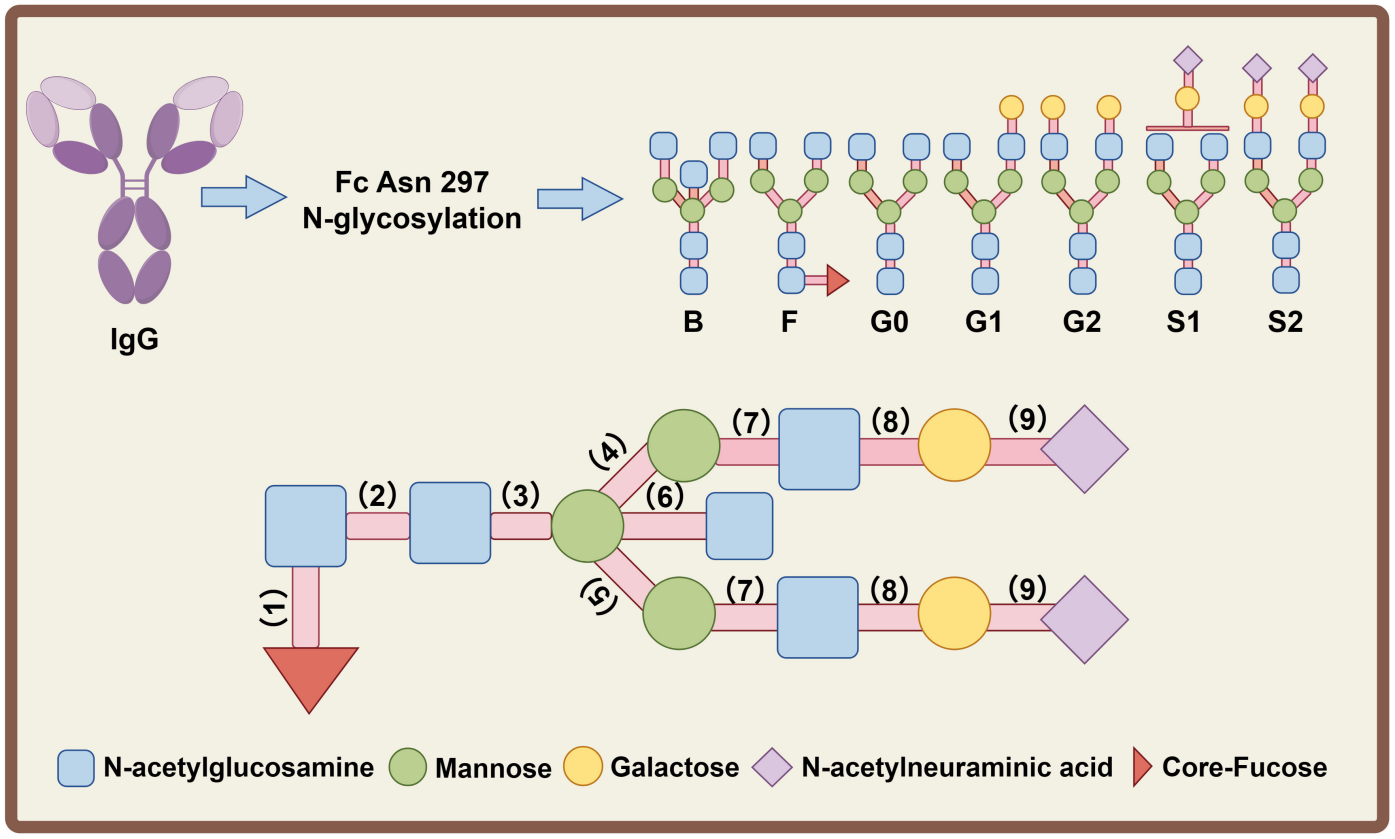
The structural composition of N-glycans at asparagine 297 (Fc Asn 297) of IgG. All glycans share a conserved core pentasaccharide (2 N-acetylglucosamine + 3 mannose); sugar residues are denoted as: N-acetylglucosamine (light blue square), mannose (green circle), galactose (yellow circle), sialic acid (N-acetylneuraminic acid, purple diamond), core-fucose (red arrow). Glycoform abbreviations: B (bisecting N-acetylglucosamine), G0 (agalactosylated), G1 (monogalactosylated), G2 (digalactosylated) for terminal galactosylation; S1 (monosialylated), S2 (disialylated); F (core fucosylated). Glycosyltransferases (1)-(9): (1) α-1,6-fucosyltransferase (Fut8), (2) β-1,4-N-acetylglucosaminyltransferase, (3) β-1,4-mannosyltransferase I, (4) α-1,3-mannosyltransferase VI, (5) α-1,6-mannosyltransferase VIII, (6) β-1,4-N-acetylglucosaminyltransferase III (GnT-III), (7) N-acetylglucosaminyltransferase (GnT-I/II), (8) β-1,4-galactosyltransferase, (9) α-2,6-sialyltransferase I or α-2,3-sialyltransferase IV/VI. These structures reveal the conserved backbone and modification diversity of IgG Fc N-glycans.

The conserved pentasaccharide core of IgG N-glycans consists of three mannose (Man) residues and two N-acetylglucosamine (GlcNAc) residues. Within the Golgi apparatus, mannosidases trim excess Man residues from the core glycans, laying the foundation for subsequent branch (antenna) formation (38). N-acetylglucosaminyltransferases (GnTs) catalyze the attachment of GlcNAc to the trimmed Man residues, generating a biantennary core precursor. For the addition of a bisecting GlcNAc, β1,4-linked N-acetylglucosamine is attached to the core β-mannose residue (the central Man residue linking the two branch Man residues in the pentasaccharide core). The biantennary core precursor can be further extended with galactose (Gal) and N-acetylneuraminic acid (Neu5Ac) on the antenna GlcNAc residues, while core fucose (Fuc) is attached to the innermost GlcNAc residue of the pentasaccharide core, together forming complex biantennary N-glycan structures (**Figure 2**) (39). The structure of IgG N-glycans is highly heterogeneous due to variations in glycan chain length, antenna number, and monosaccharide modification. Human serum IgG predominantly harbors complex N-glycans with biantennary (two branches) structures, with minor populations of triantennary (three branches) or tetraantennary (four branches) variants, whereas IgG Fc fragments exclusively contain complex biantennary N-glycans. These Fc-associated biantennary N-glycans exhibit more than 30 potential structural combinations, driven by differences in galactosylation, sialylation, core fucosylation and bisecting GlcNAc (40).

The IgG Fc domain enhances effector functions by interacting with Fcγ receptors (FcγRs) on myeloid cells and NK cells, or by initiating complement activation. IgG antibodies are classified into four subclasses (IgG1–IgG4) based on amino acid sequence differences in their heavy chain constant regions, with each subclass exhibiting distinct binding affinities for members of the FcγR family (FcγRI–FcγRIII) (41). Importantly, IgG N-glycosylation modulates these effector functions by regulating the binding affinity of the Fc domain for FcγRs or complement component C1q, including ADCC, complement-dependent cytotoxicity (CDC), and antibody-dependent cell phagocytosis (ADCP) (42).

## 4 Altered IgG Fc N-glycosylation in virus infections and underlying mechanisms

**Figure 3:**
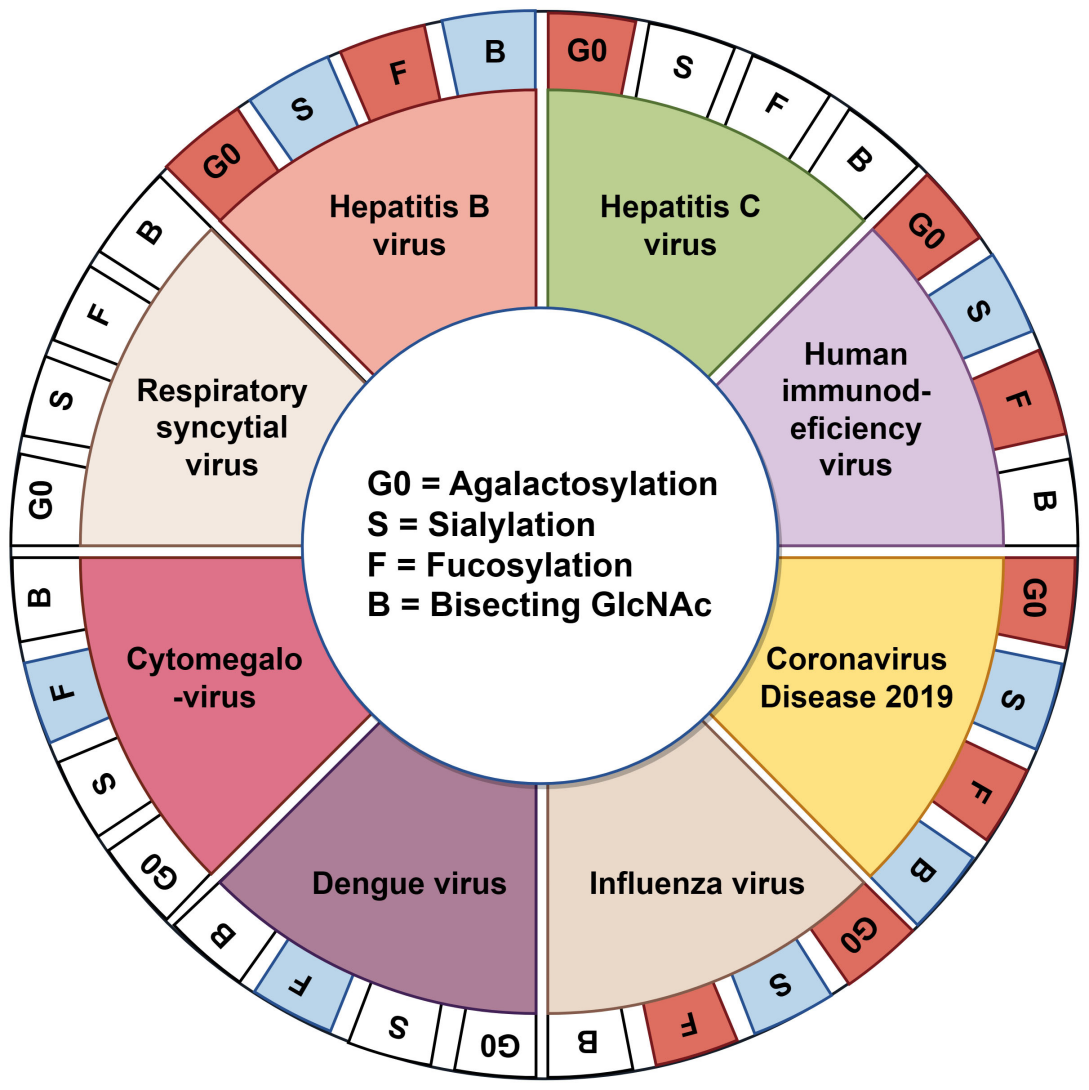
Alterations in IgG glycome traits are linked to diverse pathological conditions. The inner circle defines abbreviations for N-glycan derived traits (G0, S, F, B). The middle circle represents reported viral infectious diseases with documented IgG N-glycan derived traits. The outer circle illustrates the corresponding trends of IgG Fc N-glycan modifications. Red, increase; blue, decrease; white, no change or unreported.

**Figure 4:**
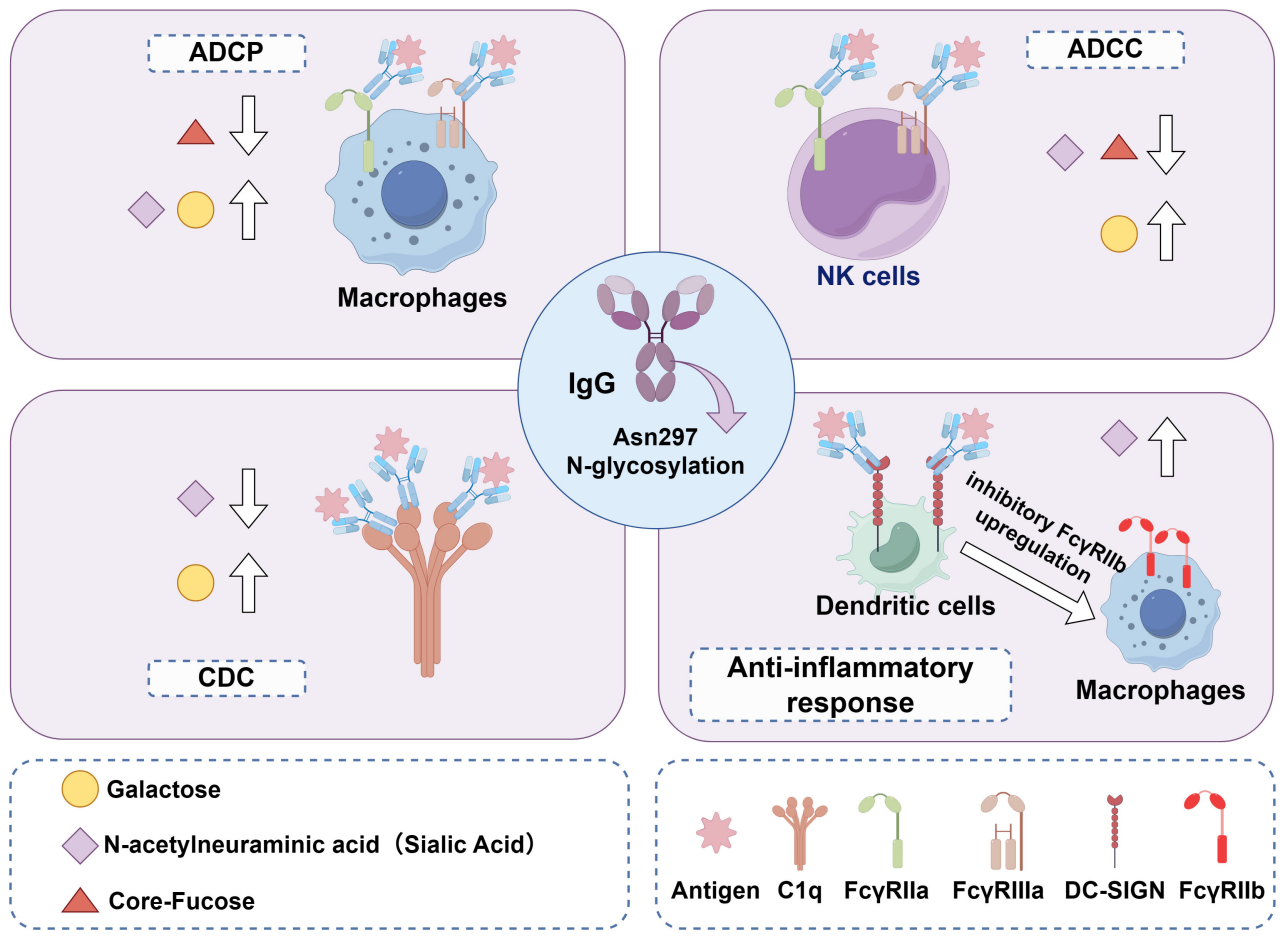
IgG effector functions dictated by Fc Asn 297 N-glycan composition. This schematic shows how distinct Fc N-glycosylation patterns (core fucose, terminal galactose/sialic acid) regulate IgG binding to immune cell receptors or complement components, thereby modulating key effector activities: ADCP (macrophages); Enhanced by reduced core fucose and increased galactose via FcγRI binding. ADCC (NK cells): Promoted by decreased core fucose and elevated galactose, mediated by FcγRIIIa engagement. CDC; Strengthened by increased galactose through IgG Fc binding to complement C1q. Anti-inflammatory response, Facilitated by elevated sialic acid on IgG Fc via binding to inhibitory FcγRIIb on dendritic cells/macrophages.

While some studies have reported alterations in serum IgG N-glycosylation in patients with certain viral infections, the precise mechanisms underlying these changes remain largely unclear. Furthermore, it has not yet been determined whether there are consistent patterns and correlations in N-glycosylation alterations induced by different viral infections. In this study, we reviewed IgG N-glycosylation alterations associated with various viral infections (**Figure 3**), focusing on changes in galactosylation, sialylation, fucosylation and bisecting GlcNAc as well as their underlying causes (**Figure 4**). Additionally, we evaluated the potential of these glycosylation alterations as biomarkers for the early diagnosis and prognosis of viral diseases.

### 4.1 Galactosylation

Numerous viral infections can induce alterations in serum IgG galactosylation in patients. HBV and HCV are major pathogens causing severe liver diseases such as chronic hepatitis, cirrhosis, and hepatocellular carcinoma (HCC) (43). A study of 299 adults demonstrated that patients with chronic hepatitis B (CHB) and HBV-related cirrhosis exhibited increased agalactosylation (G0) in the IgG1 Fc region, and decreased monogalactosylation (G1) and digalactosylation (G2) levels. Interestingly, after 48 weeks of nucleoside analogue treatment, these glycosylation alterations were reversible in CHB patients but not in those with cirrhosis (44). Consistent with these findings, another study involving 334 subjects—including healthy controls, patients with HCV infection at different disease stages, liver cirrhosis complicated with HCC group and those with non-HCV-related cirrhosis—also detected a marked increase in galactose-deficient anti-gal IgG levels (45). A study enrolling 90 participants demonstrated that individuals with HIV infection present significantly elevated serum levels of the G0, concurrent with decreased concentrations of the G1 and G2 (46). An investigation of 26 samples demonstrated that HIV-infected individuals with unsuppressed viral replication exhibited significantly elevated plasma IgG G0 levels and decreased G2 levels compared with healthy controls and HIV-infected patients with virological suppression on antiretroviral therapy (ART). Further analysis within the ART-suppressed group identified significant negative correlations between IgG G1/G2 levels and HIV DNA/RNA loads (47). In another study involving 47 ART-suppressed HIV-infected individuals, G2 levels correlated with a prolonged time to viral rebound; notably, G2 levels at the time of rebound following treatment interruption (TI) and at the viral set point were significantly lower than those measured prior to TI (48). Additionally, an increase in G0 and a decrease in G2 were observed in a cohort of 56 children with vertically transmitted HIV infection. Notably, this study also found that the glycosylation of p24-specific IgG in HIV-infected children is consistent with that of total IgG, whereas gp120-specific IgG shows a high level of G0 (49). Furthermore, the level of G0 in HIV-specific antibodies (gp120) followed a similar upward trend as total IgG but was elevated to a greater extent in adult HIV-infected individuals (46).

In the context of severe acute respiratory syndrome coronavirus 2 (SARS-CoV-2) infection, an analysis of serum IgG Fc N-glycosylation profiles in 82 unvaccinated patients within 72 hours of diagnosis revealed a significant association between COVID-19 and IgG N-glycosylation. Compared with healthy individuals, patients with moderate to severe COVID-19 exhibited elevated G0 levels and reduced G2 levels (50). Two additional studies with sample sizes of 154 and 197, respectively, corroborated these findings, reporting increased G0 levels and decreased G1 and G2 levels (51, 52). Furthermore, a study involving 292 COVID-19-infected individuals identified a reduction in total IgG1 galactosylation levels (53). Meanwhile, two separate studies, encompassing 104 and 125 COVID-19 patients respectively, demonstrated that in comparison to patients with mild symptoms, those with severe illness exhibited significantly elevated G0 levels and concomitantly reduced G2 levels (54, 55). In addition, Delbo Larsen et al. also observed a slight decrease in galactosylation levels in severe COVID-19 patients, but in their study, they attributed this decrease to aging (56). However, studies by Kedengren et al. (n = 202) found no significant differences in IgG N-glycan galactosylation levels between COVID-19 patients and healthy controls (57).

Dynamic changes in IgG galactosylation have also been documented in influenza virus infection. A study of 120 participants demonstrated that the overall IgG G0 and G2 levels in patients remained stable within 1–28 days after influenza virus infection, but a significant increase in G1 levels was observed during the disease progression period (7–28 days) (52). It remains unclear whether dengue virus infection induces alterations in total IgG galactosylation levels. Furthermore, a large-scale study of 1,826 participants reported that serum IgG1 galactosylation levels remained unchanged in individuals infected with cytomegalovirus (CMV) (58). The impact of respiratory syncytial virus (RSV) infection on serum total IgG N-glycosylation has only been studied in infants, with no observed changes in galactosylation levels (59). Nevertheless, increased IgG G0 has been observed in the majority of viral infections.

The exact mechanism underlying the reduction in IgG galactosylation remains incompletely understood, but it may be linked to elevated β-galactosidase activity and diminished β-1,4-galactosyltransferase 1 (B4GALT1) activity (60, 61). Previous animal studies have demonstrated that specific targeting of B4GALT1 in B cells can attenuate HCC progression by decreasing serum IgG galactosylation and altering associated glycan structures (62). IgG galactosylation exerts bidirectional regulatory effects on inflammatory responses by modulating key antibody effector functions, including ADCC, ADCP, and CDC. Increased IgG G1 and G2 enhance their binding affinity for FcγRIIa and FcγRIIIa, thereby improving ADCC and ADCP efficacy, and for C1q, which enhances CDC activity (63–65). A subsequent study demonstrated that IgG G0 exposes mannose-rich core glycan structures, thereby enhancing binding to mannose-binding lectin (MBL) and eliciting pro-inflammatory responses (66).

Sialic acid attaches to biantennary glycan structures terminating in one or two galactose residues; thus, agalactosylated IgG lacks sialic acid and exhibits high pro-inflammatory activity (32, 67). Additionally, IgG1 immune complexes (IgG1 IC) promote the association between FcγRIIb and dectin-1, a complex formation that inhibits C5aR-mediated ERK1/2 phosphorylation and C5a-dependent inflammatory responses. Importantly, increased IgG galactosylation is critical for the inhibitory effects of IgG1 IC, contributing to its anti-inflammatory properties (68). Conversely, in severe COVID-19 cases, lower IgG galactosylation levels induce stronger NK cell activation and higher secretion of IFN-γ and TNF-α, indicating the enrichment of pro-inflammatory IgG glycoforms and enhanced immune activation, which correlate with poor disease outcomes (50). Effective antiviral treatment in patients with CHB can reverse these abnormal IgG glycosylation patterns, reflecting the dynamic nature of IgG glycosylation during treatment. Therefore, viral infection-induced alterations in IgG galactosylation are closely associated with the pro-inflammatory or anti-inflammatory responses of the host, thereby regulating the host’s immune response against viruses.

### 4.2 Sialylation

In contrast to the alterations observed in the G0, IgG sialylation levels are markedly reduced in the majority of viral infectious diseases. For instance, compared with healthy controls, patients with CHB and HBV-related cirrhosis exhibit a significant reduction in IgG1 sialylation. Notably, this reduction in sialylation can be reversed in CHB patients following 48 weeks of treatment with nucleoside analogs for HBV (44). It remains unclear whether there is a difference in sialylation levels between HCV-infected individuals and healthy people. In a study examining HCV/HIV co-infection, which included groups with HCV monoinfection (n=14), HIV/HCV co-infection with antiretroviral therapy (ART)-mediated virological suppression (n=27), and HIV monoinfection with ART-mediated virological suppression (n=23), patients with HCV monoinfection had significantly lower IgG sialylation levels compared to those with HIV monoinfection under ART suppression (69). Moreover, HIV-infected individuals demonstrate reduced IgG disialylation (S2), with the lowest levels observed in those with unsuppressed viral replication compared to ART-suppressed patients (47). Additionally, decreased sialylation correlated with a prolonged HIV rebound time (48). Interestingly, no significant sialylation alterations have been observed in pediatric patients with vertically transmitted HIV infection. Even so, in HIV-infected children, gp120-specific IgG exhibits high sialylation (49). Meanwhile, the level of sialylation in HIV-specific antibodies (gp120) in HIV-infected adults was lower compared to that in total IgG (46).

Decreased sialylation in COVID-19 patients has also been confirmed in two studies (51, 52). Moreover, compared with patients with mild COVID-19, the level of sialylation in patients with severe COVID-19 decreased (54–56). Furthermore, Delbo Larsen et al. still attribute this decline to aging. Notably, research by Vicente, M. M et al. demonstrated reduced sialylation levels in IgG2 and IgG3, whereas studies by Siekman, S. L et al. indicated no changes in IgG1 sialylation (50, 53). These findings tentatively suggested that the overall decrease in IgG sialylation levels might stem primarily from changes in IgG2 and IgG3. In influenza virus infection, sialylation dynamics exhibit a distinct temporal pattern: no change was observed during the initial 1–7 days post-infection, followed by a decline from 7 to 28 days post-infection (52). However, sialylation alterations in dengue virus infections have not yet been investigated. Consistent with previous findings on galactosylation, serum IgG1 sialylation remains unchanged in individuals infected with CMV (58). The impact of RSV infection on serum total IgG N-glycosylation has only been studied in infants, with no observed changes in sialylation levels (59). Sialylation tends to decrease in most viral infectious diseases.

In most cases, sialylated IgG acts as a mediator of anti-inflammatory processes. IgG sialylation is believed to increase binding affinity for dendritic cell-specific ICAM-3-grabbing non-integrin (DC-SIGN), enhance the gene expression of the inhibitory FcγRIIb, and promote anti-inflammatory processes (70). In viral infections, decreased sialylation enhances IgG’s affinity for FcγRIIIa on NK cells, thereby boosting ADCC and pro-inflammatory effects (71). This is likely because sialic acid reduces antibody hinge flexibility, thereby lowering receptor binding. Sialylation also reduces the affinity of galactosylated IgG for C1q, suppressing CDC and exerting anti-inflammatory effects (72). Additionally, higher sialylation enhances IgG’s affinity for FcγRIIa, boosting ADCP, and for FcRn, extending IgG’s half-life (73, 74). Viral infection may induce inflammation by downregulating IgG sialylation and promoting the release of inflammatory factors.

### 4.3 Fucosylation

In prevalent viral infections, including HBV, HCV, HIV, and COVID-19, most studies have reported a consistent trend of decreased galactosylation and sialylation levels; however, the alterations in fucosylation exhibit variability across these infections. In a study with a sample size of 745, IgG core fucosylation was observed in the serum of patients with HBV-related HCC (75). It is still unclear whether there is a difference in fucosylation between HCV-infected individuals and healthy people. Currently, only one study on HCV and HIV co-infection has demonstrated that the levels of fucosylation in the HCV mono-infection group and the co-infection group are higher than those in the HIV mono-infection group (69). Significantly increased fucosylation levels were found in HIV-infected adults (47). Among HIV-infected children, the level of fucosylation was higher in progressors compared to non-progressors. Meanwhile, gp120-specific IgG in HIV-infected children exhibits high fucosylation (49). Furthermore, fucosylation levels were closely associated with a prolonged viral rebound time (48).

Three studies have found that the serum IgG fucosylation level was elevated in COVID-19 patients, and the elevation was more significant in severe cases (51, 52, 54, 55). Conversely, four studies also found no change in serum IgG fucosylation levels in COVID-19 infected individuals (50, 53, 56, 57). Moreover, one study further revealed that the anti-S antibody IgG1 in patients with severe COVID-19 was mostly in the afucosylated form, whereas the fucosylation level of anti-S antibody IgG1 in patients with mild cases was normal. Additionally, the fucosylation level of anti-N antibody IgG was generally high, with only a mild decrease in fucosylation observed in severe patients (56). Similarly, IgG fucosylation in influenza virus-infected individuals also exhibits a distinct temporal pattern: fucosylation levels decrease within the first 1–7 days post-infection and then increase from 7 to 28 days post-infection (52). On the other hand, studies have shown that IgG1 fucosylation levels were decreased in dengue virus-infected individuals compared to those infected with Zika virus (ZIKV) and West Nile virus (WNV). Moreover, the level of antigen-specific IgG1 fucosylation in patients infected with the dengue virus also shown a decrease (76, 77). Furthermore, CMV infection significantly reduced IgG1 fucosylation (58). As for RSV infection, its impact on serum total IgG N-glycosylation has only been studied in infants, with no observed changes in fucosylation levels (59). The aforementioned reports suggest that, in contrast to galactosylation and sialylation, alterations in fucosylation exhibit variability across different viral diseases.

The FUT family consists of fucosyltransferases, among which FUT8 is the sole enzyme responsible for core fucosylation of mammalian N-glycoproteins (78). Studies have indicated that FUT8 is highly expressed in HBV- and HCV-related HCC, and affects tumor progression (79, 80). FUT8 upregulation in HCV-infected cells enhances the binding of Lens culinaris agglutinin (LCA) to fucosylated ANXA2 and HSP90B (81). Furthermore, FUT8 expression correlates with tumor size, and its knockdown inhibits the proliferation, migration, and invasion of MHCC97-H (a liver cancer cell line) (82). FUT8 is also involved in the expression of viral envelope proteins, such as SARS-CoV-2 spike protein and HIV gp120. Silencing FUT8 enhances the antiviral IFN-I response and inhibits RNA virus replication, indicating a crucial role of FUT8 in the host’s innate immune defense against viruses (83). Therefore, viral infection-induced upregulation of FUT8 may explain the elevated IgG core fucosylation observed in some virus-infected patients.

In contrast, certain viruses may reduce the fucosylation of host proteins (including IgG) or their own envelope proteins through structural adaptations and immune evasion strategies. Reduced IgG core fucosylation can enhance antibody-NK cell interactions, boost ADCC and moderately promote ADCP by enhancing binding to FcγRIIIa on NK cells and FcγRIIa on phagocytes (84, 85). Additionally, reduced core fucosylation enables IgG to bind to Fcγ receptors on monocytes, inducing the secretion of pro-inflammatory cytokines including IL-1β, IL-6, TNF-α, and IFN-γ, and promoting hepatic synthesis of C-reactive protein (CRP), a key acute-phase protein, leading to pro-inflammatory responses. In a mother-infant cohort study, it was further demonstrated that ADCC activation mediated by IgG with reduced core fucosylation was associated with a significantly reduced risk of intrauterine human CMV transmission (86). However, core fucosylation alterations have minimal impact on CDC, as the core fucose residue is spatially distant from the C1q-binding domain of the IgG Fc region (87).

### 4.4 Bisecting GlcNAc

Similar to fucosylation, the patterns of bisecting GlcNAc modification exhibit significant heterogeneity across different viral species. In HBV infection, the level of bisecting GlcNAc was significantly reduced, but it can also be reversed after treatment (44). It is unclear whether the level of bisecting GlcNAc in total serum IgG of HIV-infected individuals is different from that of healthy people. Currently, only one study conducted in children has shown that the level of bisecting GlcNAc in total IgG was lower in non-progressors compared to progressors, with levels in gp120-specific IgG being even lower (49). Compared with healthy individuals, a decrease in bisecting GlcNAc was observed in COVID-19 infected patients (51–54). However, there are varying opinions regarding the differences in bisecting GlcNAc between severe and mild COVID-19 (50, 54–56). In patients with influenza virus infection, bisecting GlcNAc levels decline during the first 1–7 days post-infection, whereas remained stable from day 7 to day 28 (52). In contrast, bisecting GlcNAc levels remain unaltered in individuals infected with CMV (58). The impact of RSV infection on serum total IgG N-glycosylation has only been studied in infants, with no observed changes in bisecting GlcNAc levels (59). To date, whether bisecting GlcNAc levels are altered in HCV and dengue virus infections has not been investigated.

Despite the well-established role of bisecting GlcNAc as a key regulator of IgG Fc-mediated effector functions, controversies remain regarding the precise molecular mechanisms underlying its functional impacts. The prevailing model in the field supports that bisecting GlcNAc predominantly exerts its effects through an indirect regulatory mechanism, rather than direct modulation of IgG-Fcγ receptor interactions, although a small subset of studies has proposed potential direct effects on Fc conformation or receptor binding (88). Specifically, the addition of bisecting GlcNAc to the conserved N-glycosylation site at Asn297 of the IgG Fc domain sterically hinders access of FUT8 to the glycan core, thereby inhibiting the addition of core fucose residues to the N-glycan structure (78). This reduction in core fucosylation, in turn, significantly enhances the affinity of IgG for activating FcγRIIIa receptors, ultimately leading to the augmentation of ADCC and ADCP effector functions.

## 5 IgG N-glycosylation as a potential diagnostic biomarker in viral infection-associated diseases

Currently, viral nucleic acid detection is the standard method for identifying viral infections. While accurate, this method cannot track disease progression or predict clinical outcomes (89). Given the alterations in IgG N-glycosylation patterns during viral infections, numerous researchers have investigated IgG N-glycosylation-related indicators and explored their potential as diagnostic and prognostic biomarkers. Once IgG N-glycosylation enters the circulation, it remains stable (except for sialylation), which reflects the host’s immune status and provides valuable information for the diagnosis and prognostic evaluation of related diseases (90). Thus, IgG N-glycosylation biomarkers may serve as promising diagnostic and prognostic indicators.

For instance, HBV and HCV carriers are at high risk of developing cirrhosis and HCC. Liver biopsy is the gold standard for diagnosing fibrosis stages but is invasive, which deters many patients. The diagnostic efficacy of alpha-fetoprotein (AFP) and protein induced by vitamin K absence or antagonist-II (PIVKA-II) in early HCC is limited. A study involving 760 patients with CHB revealed that serum IgG N-glycans can be used for non-invasive diagnosis of liver fibrosis. In this study, 7 peaks (NGA2F, NGA2FB, NG1A2F, NA2, NA2FB, NA3, NA4Fb) were selected from differential N-glycan peaks through LASSO regression to construct the Px model. The results showed that the Px model was significantly superior to liver stiffness measurement, aspartate aminotransferase to platelet ratio index, and fibrosis - 4 index (AUC = 0.766) (91). Serum IgG L3% (IgG with high Lens culinaris agglutinin (LCA) affinity and high fucosylation) exhibited a diagnostic accuracy of 81.3% in distinguishing HCC from non-HCC, surpassing AFP’s 78.0%. It also outperformed AFP in differentiating liver cirrhosis (LC) from HCC, suggesting its potential as a diagnostic biomarker. Additionally, serum IgG L3% could serve as a prognostic indicator, as higher levels in HCC patients correlate with poorer prognosis and increased postoperative recurrence (75). Furthermore, IgG N-glycan A2G1 (6) FB accurately distinguishes HCC from LC (area under the curve (AUC) = 0.9614) (92). The N-linked glycans of anti-Gal IgG in healthy individuals were mainly FcA2G2 (core fucosylated biantennary, containing 2 galactoses). In patients with HCV infection-related liver cirrhosis, the anti-Gal IgG was mainly FcA2G0 (core fucosylated biantennary, without galactose). Galactose-deficient anti-Gal IgG could be used as a marker for liver fibrosis/cirrhosis (AUC = 0.93), and its efficacy was better than some traditional indicators (such as serum γ-globulin) (45).

Following HIV infection, IgG N-glycans can track disease progression, including the time to HIV rebound during ART, which is associated with IgG G2 levels. IgG galactosylation and sialylation patterns can predict COVID-19 progression, with higher G0 levels often indicating poor prognosis (48). Specifically, IgG G2, IgG1 G0, and IgG2&3 G2 can independently predict patient prognosis (AUC = 0.722, 0.714, and 0.705, respectively), and their combination significantly improves the prediction of COVID-19 progression (AUC = 0.729) (50). In dengue virus infection, reduced IgG1 fucosylation predicted disease severity. This association was supported by the finding that patients who developed severe manifestations, including dengue hemorrhagic fever and dengue shock syndrome, displayed significantly elevated levels of heightened levels of afucosylated IgG1 (76). Additionally, the fucosylation status of maternal anti-dengue IgG predicted infant susceptibility in a separate study (77). These IgG glycan-related indicators show good sensitivity and specificity, indicating their potential as diagnostic and prognostic biomarkers.

## 6 Conclusions

Viral infections can stimulate the host immune system to elicit a response, and the activation of immune effector mechanisms such as ADCC, ADCP, and CDC is closely associated with the composition of IgG Fc N-glycans. Following viral infection, alterations in IgG N-glycosylation are observed, typically characterized by decreased galactosylation and sialylation; however, changes in fucosylation and bisecting GlcNAc exhibit a more complex pattern. The underlying mechanisms driving these changes warrant further investigation. Additionally, accumulating evidence suggests that IgG N-glycosylation alterations may serve as biomarkers for tracking the progression of various viral diseases and predicting their severity. Consequently, the analysis of serum IgG N-glycosylation changes in patients may provide novel insights for the diagnosis and treatment of viral infection-associated diseases.
